# Respiratory sensitivity is reduced in functional neurological disorder and associated with higher somatoform dissociation

**DOI:** 10.1093/braincomms/fcaf283

**Published:** 2025-07-31

**Authors:** Natascha Stoffel, Petr Sojka, Nicolas Gninenko, Michael Mouthon, Laure von der Weid, Tereza Serranová, Selma Aybek

**Affiliations:** Faculty of Science and Medicine, Section of Medicine, Department of Neuro- and Movement Sciences, University of Fribourg, Fribourg 1700, Switzerland; Graduate School for Health Sciences (GHS), University of Bern, Bern 3012, Switzerland; Department of Neurology and Centre of Clinical Neuroscience, General University Hospital and First Faculty of Medicine, Charles University, Prague 12800, Czech Republic; Faculty of Science and Medicine, Section of Medicine, Department of Neuro- and Movement Sciences, University of Fribourg, Fribourg 1700, Switzerland; Faculty of Science and Medicine, Section of Medicine, Department of Neuro- and Movement Sciences, University of Fribourg, Fribourg 1700, Switzerland; Faculty of Science and Medicine, Section of Medicine, Department of Neuro- and Movement Sciences, University of Fribourg, Fribourg 1700, Switzerland; Department of Neurology and Centre of Clinical Neuroscience, General University Hospital and First Faculty of Medicine, Charles University, Prague 12800, Czech Republic; Faculty of Science and Medicine, Section of Medicine, Department of Neuro- and Movement Sciences, University of Fribourg, Fribourg 1700, Switzerland

**Keywords:** conversion disorder, interoception, respiration, dissociation, metacognition

## Abstract

Abnormal interoception—the processing of internal bodily signals—has been increasingly recognized as a key factor in the pathophysiology of functional neurological disorder. While evidence suggests reduced cardiac interoceptive accuracy in functional neurological disorder, other interoceptive domains, such as respiratory processing, remain largely unexplored. Here, we introduce a novel respiratory resistance sensitivity task to assess respiratory interoception and metacognition in functional neurological disorder. Additionally, we investigate the relationship between respiratory interoception and other interoceptive or clinical variables, including somatoform dissociation as a potential inverse correlate of interoception. Using the respiratory resistance sensitivity task, respiratory interoceptive sensitivity and metacognition were assessed, along with the response time and the decision precision for identifying the obstructed breath in the respiratory task in patients with mixed functional neurological disorder (*N* = 43) and age- and sex-matched healthy controls (*N* = 48). Drift diffusion modelling was applied to response times and discrimination decisions to assess sensory evidence accumulation. Additionally, interoceptive self-reports (multidimensional assessment of interoceptive awareness and the interoceptive accuracy scale) were collected. Associations between interoceptive measures, symptom severity, and the Somatoform Dissociation Questionnaire were analysed. Patients with functional neurological disorder showed reduced respiratory sensitivity (*P*  *=* 0.032, *d* = 0.47) and interoceptive self-report scores (*P*  *=* 0.0004, *d* = 0.79 and *P*  *=* 0.018, *d* = 0.65, respectively) compared to controls, whereas metacognition and decision precision did not differ between groups. In the functional neurological disorder group, respiratory sensitivity and metacognitive performance were negatively associated with somatoform dissociation scores (*r* = −0.38, *P* = 0.011 and *r* = −0.36, *P* = 0.017, respectively). While no group difference was found for the response time, we did identify a negative correlation with response time and respiratory sensitivity (*r* = −0.27, *P* = 0.013) and reduced drift rate in patients with 89% posterior probability. Further, perceived breathlessness (*r* = −0.24, *P* = 0.026) was negatively associated with the task performance. This study provides first evidence of impaired respiratory interoception in patients with functional neurological disorder. We were able to demonstrate a moderate-sized group difference in a large cohort, using a valid respiratory task, that is, associated with clinical variables such as self-reported severity of somatoform symptoms. Further, reduced drift rates for patients with functional neurological disorder indicated less efficient sensory evidence accumulation, while indifferent boundary separation indicated preserved decision caution. These novel insights into respiratory interoception in functional neurological disorder suggest it may represent a therapeutic target for future investigation.

## Teaser

Stoffel and Sojka demonstrated that respiratory sensitivity was lower for patients with functional neurological disorders, along with reduced interoceptive sensibility but intact metacognition, compared to healthy controls. Both respiratory sensitivity and metacognition was further negatively associated with somatoform dissociation, highlighting the involvement of interoception in the disorder.

## Graphical Abstract

**Graphical Abstract:**
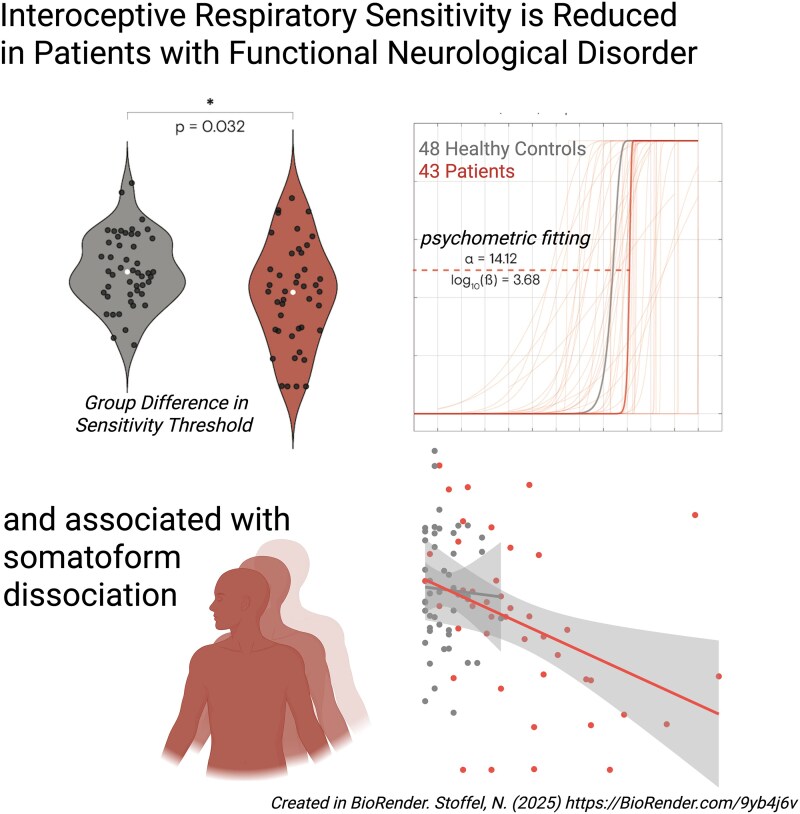

## Introduction

Functional neurological disorder (FND) is a common neuropsychiatric condition that manifests as a wide range of neurological symptoms including seizures, abnormal movements, weakness, dizziness, sensory and cognitive symptoms that reflect abnormal communication within and between brain network.^1^ The resulting disability is caused by both the core clinical presentation and additional symptomatology across body systems such as chronic pain, fatigue and psychological symptoms including depression, anxiety and dissociation. Functional symptoms may arise from the same neurobiological vulnerabilities and share a common underlying pathophysiology.

The events that trigger FND are diverse and include almost any unexpected change in the physiology. From a psychiatric perspective, emphasis has been placed on adverse life experiences^2,3^ and emotional distress^4,5^ as precipitating factors or acute triggers in FND. In neurology, bodily events such as physical injury,^6,7^ acute illness,^8^ vaccination^9^ or drug side effects^1^ are increasingly recognized as potential triggers. These perspectives converge on a pathophysiological model suggesting that bodily perturbations, combined with difficulties in discriminating changes in bodily states, may lead to the misattribution of benign bodily sensations (e.g. physical fatigue from exercise or emotional arousal) as harmful.^10^ These misattributions are thought to manifest as symptoms through active inference mechanisms.^11^

Over the last decades, interoceptive dysfunction has been discussed concerning a range of neuropsychiatric disorders, including FND.^12^ Interoception is both a conscious and an unconscious process that involves sensing, integrating, and interpreting signals from the inside of the body.^13^ However, inconsistent definitions and measurements prompted the development of a multi-dimensional model with interoceptive accuracy, self-report and insight (metacognition) representing the three most commonly measured dimensions of consciously accessible interoception.^14^ ‘Interoceptive accuracy’ refers to objective performance, typically measured using tasks that assess the ability to detect interoceptive signals, such as heartbeats. ‘Interoceptive self-reports’ assess one’s self-perceived interoceptive ability (e.g. the beliefs concerning one’s interoceptive sensations and experiences via questionnaire-based scales). Finally, ‘interoceptive insight or metacognition’ is derived from self-reported confidence judgments about a task-specific performance, yielding a performance-independent score that reflects the degree to which interoceptive accuracy is predicted by subjective confidence in one’s task performance.^15^

Experimental research on interoception is challenging because controlling the intensity of interoceptive signals is often invasive and infeasible. Consequently, most studies on interoceptive accuracy in FND have relied on heartbeat counting tasks (HCT) where subjects monitor heart sensations while at rest. Some evidence has shown that patients with FND have lower cardiac interoceptive accuracy,^16-19^ but not all studies support this finding or only show group differences in the dimensions of self-report or metacognition.^20-23^ Neurophysiological^24,25^ and neuroimaging evidence,^22^ although limited, supports abnormal processing of cardiac inputs in FND, underscoring altered interoception as an important feature in this condition. In summary, these findings suggest that the reduced ability to process interoceptive inputs in FND should be further empirically tested.

The validity of heartbeat detection tasks has been questioned on both conceptual and methodological grounds.^26^ In particular, interoceptive accuracy measured by the HCT may primarily reflect under-reporting of heartbeats, as detecting heartbeats is inherently challenging.^26^ As a result, performance on this task is heavily influenced by non-interoceptive factors, such as individuals’ beliefs about their heart rate.^27^ These findings raise concerns, especially in light of the growing interest in using interoceptive measurements as potential biomarkers for neuropsychiatric conditions.^27^

To extend the evidence for impaired interoception in FND beyond the cardiac domain, we used a respiratory resistance sensitivity task (RRST), a novel psychophysical approach to measure patients’ sensitivity to detect subtle increases in airflow obstruction during inspiration.^28^ The RRST uses an adaptive staircase procedure to precisely control the intensity of interoceptive stimuli and to estimate the respiratory psychometric function (PMF), which relates levels of airflow obstruction to the probability of correctly discriminating between them. To capture the decision-making processes underlying performance on this task, we also applied the Drift Diffusion Model (DDM), which allowed us to dissociate perceptual sensitivity (evidence accumulation) from decision caution (boundary separation).^29^

To better understand the role of interoception in FND, this study aimed to empirically investigate the ability to process interoceptive respiratory inputs in FND compared to healthy controls (HCs) using the RRST. We hypothesized that these interoceptive characteristics, i.e. the ability to discriminate between interoceptive stimuli or to correctly estimate one’s perceptual capacity (metacognition), are reduced in patients with FND and are associated with examiner-rated symptom severity and self-rated symptoms of somatoform dissociation.

## Materials and methods

### Participants

A total of 92 subjects participated in the study targeting a comprehensive assessment of interoception and biological markers in FND, registered at ClinicalTrials.gov (NCT06084325), for which *N* = 100 participants were recruited (divided into two groups; 50 patients with FND and 50 sex and age matched controls). For the analysis of respiratory interoception, one outlier was excluded from analyses due to deviating more than 2.5 SD from the overall group mean. The final sample consisted of *N* = 43 patients with FND (31 female (72.1%); mean age = 38.5 ± 11.8 years; range = 18–64 years) and *N* = 48 controls (35 female (72.9%); mean age = 37.8 ± 13.0 years; range = 18–67 years). Patients with FND were recruited from the Psychosomatic Medicine Unit at the University Hospital/Inselspital Bern and the Clinical Neurology Unit of the Cantonal Hospital of Fribourg (HFR) and had been diagnosed with FND (including F44.4–F44.7) by a neurologist according to the Diagnostic and Statistical Manual of Mental Disorders, fifth edition (DSM-5), and to the International Classification of Diseases, 11th revision (ICD-11). Controls were recruited via flyers, word-of-mouth, and advertisements in public media. Exclusion criteria for both groups were: (i) severe comorbid psychiatric disorders (e.g. psychosis), (ii) neurological conditions (e.g. epilepsy), (iii) previous brain surgery or medical implants, (iv) history of drug or alcohol abuse, (v) cardiovascular disease and (vi) pregnancy or breastfeeding in women.

The study was approved by the local Ethics Committee of the Canton Bern (2023-00469) and conducted according to the Declaration of Helsinki. Written informed consent was provided by all subjects.

### Psychometric and clinical assessments

All participants completed two questionnaires to assess individuals’ beliefs concerning interoceptive sensations: the multi-dimensional assessment of interoceptive awareness (MAIA)^30^ and the interoceptive accuracy scale (IAS).^31^ The MAIA is a reliable and valid assessment of the tendency to focus on interoceptive signals across eight subscales (noticing, not distracting, not worrying, trusting, attention regulation, self-regulation, emotional awareness and body listening) for both clinical and non-clinical populations.^30,32^ We here evaluate the total score as a multi-dimensional score discussed to be central for interoceptive self-report,^33^ while also focusing specifically on the subscale of ‘Noticing’, which assesses whether interoceptive sensation (uncomfortable, comfortable and neutral sensations) in the body are noticed, and thus to compare this self-report between groups along the tested respiratory sensitivity.^30^ While the MAIA assesses an individual’s self-reported tendency to focus on internal sensations, the IAS assesses one’s belief in how accurately one is observing interoceptive signals.^34^

Self-reported clinical symptoms and risk factors of all participants were assessed using the Somatoform Dissociation Questionnaire (SDQ-20), evaluating aspects of sensory alterations (e.g. analgesia, numbness of body part), loss of motor control (e.g. paralysis, seizures) and pain symptoms,^35^ Beck’s Depression Inventory (BDI-II), assessing depressive symptoms,^36^ the State-Trait Anxiety Inventory (STAI), assessing both state and trait anxiety symptoms^37^ and the Childhood Trauma Questionnaire (CTQ), assessing the experience of physical, emotional and sexual abuse or neglect in childhood.^38^ The intake of psychotropic medication and the body mass index (BMI) were also recorded. All patients with FND were evaluated by the examiner for FND severity using clinical global impression (CGI)^39^ and the simplified functional movement disorders rating scale (S-FMDRS).^40^ Characteristics of the full sample are shown in Table 1, and detailed clinical characteristics of patients with FND are listed in Supplementary Table 1.

**Table 1:** Characteristics of sample

Variable	Overall, *N* = 91	HC, *N* = 48	FND, *N* = 43	*P*-value
Sex, female, count (%)	66.0 (72.5)	35.0 (72.9)	31.0 (72.1)	>0.9^a^
Age, years, median (IQR)	37.0 (30.0, 48.5)	37.0 (28.0, 50.0)	39.0 (31.5, 47.0)	0.6^b^
Intake of psychotropic medication, count (%)	21.0 (23.6)	2.0 (4.3)	19.0 (44.2)	<0.001^a^
Smoking, count (%)	15 (16)	6 (13)	9 (21)	0.4^a^
Body mass index in kg/m², median (IQR)	23.7 (21.7, 26.3)	22.7 (21.7, 25.1)	25.3 (21.8, 27.8)	0.019^b^
BDI-II, median (IQR)	8.0 (3.0, 15.0)	4.0 (1.0, 8.0)	15.0 (9.5, 23.5)	<0.001^b^
STAI-T, median (IQR)	41 (32, 49)	37 (30, 45)	46 (38, 55)	<0.001^b^
CTQ, median (IQR)	37.0 (30.0, 54.5)	34.0 (29.0, 41.3)	46.0 (35.0, 60.5)	0.001^b^
SDQ-20, median (IQR)	27.0 (22.0, 36.0)	23.0 (21.0, 26.0)	36.0 (28.5, 45.0)	<0.001^b^

^a^Pearson’s χ^2^ test.

^b^Mann–Whitney U-test.

BDI-II, Beck’s depression inventory II; STAI-T, Spielberger’s trait anxiety inventory; CTQ, Bernstein’s childhood trauma questionnaire; SDQ-20, Nijenhuis’ Somatoform Dissociation Questionnaire; IQR, inter-quartile range.

### Respiratory interoception

Respiratory interoception was measured using the RRST, developed by Nikolova *et al*.^28^ Full details of the device assembly and task setup can be found in the original publication.

**Figure 1:**
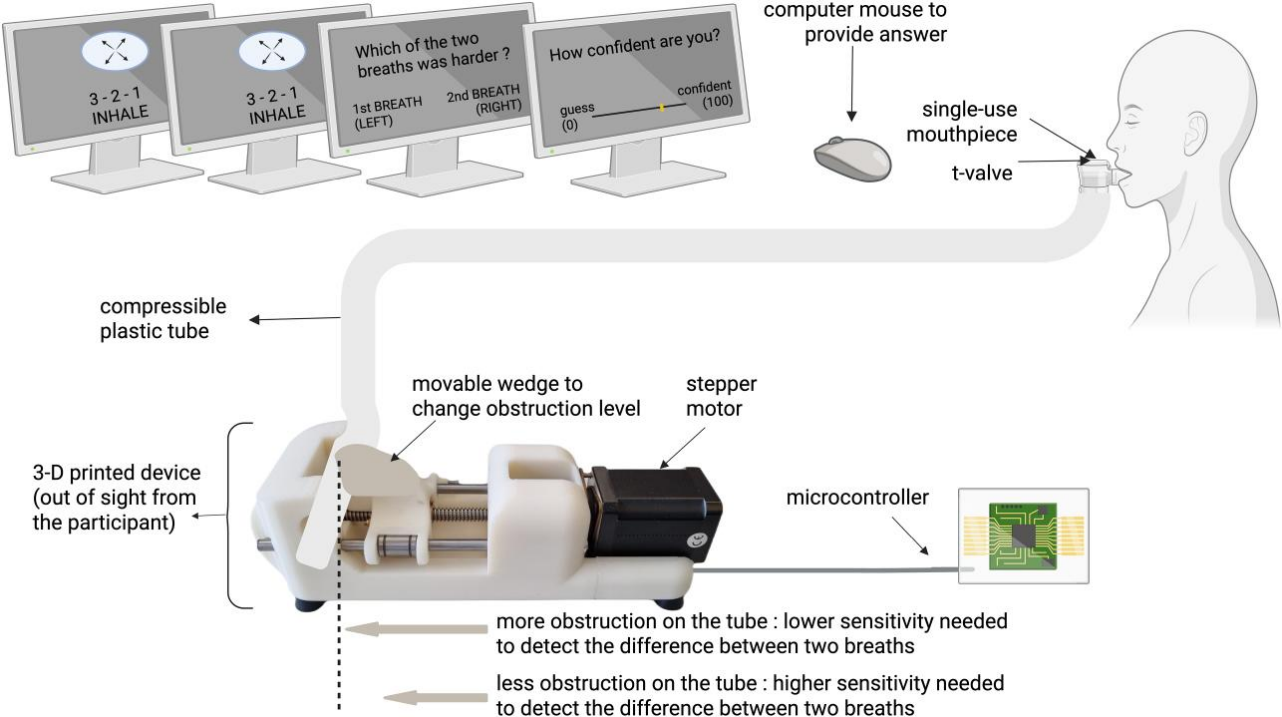
**Set up of respiratory resistance sensitivity task (RRST).** Illustration of the task setup together with the RRST device. The device was 3D-printed according to Nikolova *et al*.,^28^  https://doi.org/10.1016/j.biopsycho.2022.108325. Illustration was created in BioRender. Stoffel, N. (2025) https://BioRender.com/n42d329.

In brief, a breathing tube was connected to a computer-controlled device that varied the degree of airflow obstruction using a psi staircase algorithm. During the task, participants took two sharp inhalations (as opposed to deep, slow inhalations) through disposable mouthpieces with antibacterial filters, connected to the tube via a low dead space T-valve. Each trial consisted of two paced breaths (inhale = 0.8 s, pause = 0.5 s, exhale = 3 s) guided by a visual cue, with the obstruction randomly applied to either the first or second inhale. In a two-alternative forced-choice (2AFC) format, participants decided which of the two breaths felt more obstructed and indicated their confidence using the computer mouse on a visual scale shown on the screen within 6 s. The task setup is shown in Fig. 1.

Participants first completed a practice round of six trials to familiarize themselves with the task. Sensitivity measurements were then performed over three blocks of a total of 50 trials, with a minimum two-minute break in between blocks and inter-trial interval of 3.5 to 4 s. The individual sensitivity threshold was determined by fitting a Weibull PMF to identify the obstruction level at which the probability of a correct response was 75%. A lower final threshold indicated a greater sensitivity in detecting the obstructed breath. To improve readability, we subtracted this score from the maximum obstruction score (which was the maximum compression of the tube, in our case = 17) to ensure that higher scores in our results corresponded to greater sensitivity. We also computed slopes of each fitted PMF, representing the precision of interoceptive judgment. A steep slope indicates that a participant can clearly differentiate between stimuli close to the threshold. To assess metacognition—awareness of performance independent of one’s accuracy—participants indicated their confidence in discriminating between the two inhales after every trial by answering the question ‘How confident are you in your choice?’ using a visual scale with 0 = guess to 100 = certain. The metacognitive score was calculated as the area under the type II receiver operating characteristic (ROC) curve (AUC).^41^ Results on confidence-by-accuracy correlations via ordered beta regression as an alternative way of defining metacognition, are presented in Supplementary material.

In summary, our interoceptive measures derived from the RRST were respiratory sensitivity (as an indicator of interoceptive accuracy in the respiratory domain), interoceptive decision precision (derived from the slope of the fitted psychometric curve), and metascore (as a performance-independent score of confidence in the RRST).

At the end of the task, all participants rated their aversiveness, breathlessness and dizziness and reported the severity of asthma symptoms on a visual scale between 0 and 100. These control questions about the task, and response times (RTs) of a decision to detect obstruction were also analysed as a group comparison. Further, we controlled whether these ratings would affect the group differences in respiratory sensitivity by including them in a linear model as covariates.

### Drift diffusion model

To quantify latent decision-making parameters, we fitted a hierarchical DDM using the HDDM toolbox (version 0.9.8).^29^ The model decomposes RTs and choices into cognitive components including drift rate (*v*; information accumulation), boundary separation (*a*; decision caution), and non-decision time (*t*; sensory and motor delays). Lower drift rates indicate slower or less efficient accumulation of sensory evidence, reflecting poorer perceptual sensitivity, while higher drift rates reflect more accurate and efficient information processing. Lower boundary separation reflects a tendency towards faster, less cautious decisions, whereas higher values indicate more conservative decision-making, requiring more evidence before responding. A hierarchical Bayesian approach was used to estimate group- and subject-level parameters simultaneously. We compared multiple models in which *v*, *a* and *t* were selectively allowed to vary by group. Model comparison using the deviance information criterion indicated that the model allowing both *v* and *a* to vary across groups provided the best fit to the data. Posterior distributions were estimated via Markov chain Monte Carlo (MC) sampling with 10 000 samples and a burn-in of 1000 steps. Convergence was assessed via visual inspection of trace plots and MC error relative to posterior standard deviation. Parameters were considered well-estimated if MC error was <10% of the posterior standard deviation.

### Statistical analysis

Statistical analyses included Pearson’s χ^2^ test for binary variables, the Mann–Whitney U-test for continuous, non-normally distributed variables, and independent *t*-tests for continuous, normally distributed variables. Based on the previously identified association of anxiety and respiratory interoception, we also controlled the respiratory group difference for this potential confound using linear regression.^42^ Further control analysis of identified respiratory sensitivity group differences included the RT, how long in milliseconds until an answer was given identifying the obstructed breath, and the ratings of unpleasantness at the end of the task. Pearson correlations were used to assess relationships between symptom severity (SDQ-20, SFMDRS and CGI) and respiratory interoception variables (RRST sensitivity and metacognition). Significant correlations were analysed using multiple linear regression, including psychotropic medication as a covariate due to its strong association with both SDQ-20 and interoceptive measures. Secondary analyses controlled for affective symptoms (anxiety and depression sum score) as this too had marginal evidence of being a relevant covariate of no interest, and was separated per group, while the other potential covariates (age and BMI) did not show associations with both the predictor and the outcome variable and thus were not included in the model. Results on secondary analyses are reported in Supplementary Results. For all analyses, *P*-values are reported. Effect sizes are shown as Cohen’s *d* for numeric variables and odds ratios (OR) for binary variables. Pearson’s correlation coefficients are provided, with Bonferroni correction applied for multiple comparisons where necessary.

## Results

### Demographic and clinical group differences

Patients with FND exhibited a significantly higher intake of psychotropic medications (*P* = 0.0004, OR = 17.42) and BMI (*P* = 0.019, *d* = 0.56) compared to HCs. Sex and age did not differ between groups. Patients with FND demonstrated higher scores on measures of depression (*P* < 0.0001, *d* = 1.38), anxiety (state: *P* < 0.0001, *d* = 1.08, trait: *P* = 0.0002, *d* = 0.83), childhood trauma (*P*  *=* 0.001, *d* = 0.66) and somatoform dissociation (*P* < 0.0001, *d* = 1.50). These findings are summarized in Table 1. Childhood trauma and somatoform dissociation positively correlated in patients with FND (*r* = 0.33, *P* = 0.030), but not in the control population (*P* = 0.984).

### Group differences in respiratory interoception

**Figure 2:**
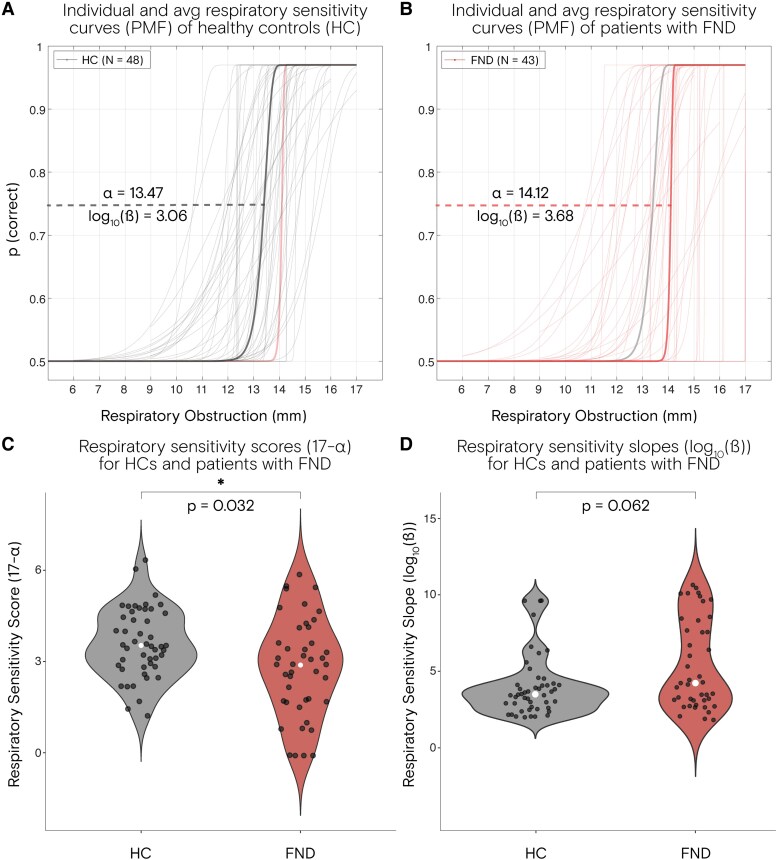
**Group differences in the respiratory resistance sensitivity task (RRST).** (**A**) Healthy controls have higher respiratory sensitivity (corresponding to 17-ɑ) compared to (**B**) patients with FND. Psychometric function (PMF) curves from the RRST are plotted for 48 HCs and 43 patients with FND. (**C**) Independent *t*-test to test for group differences in respiratory sensitivity derived from the alpha value of RRST (*P* = 0.032). (**D**) Wilcoxon test for group differences in respiratory decision precision derived from the beta value of the RRST, log transformed for better visualization.

**Figure 3:**
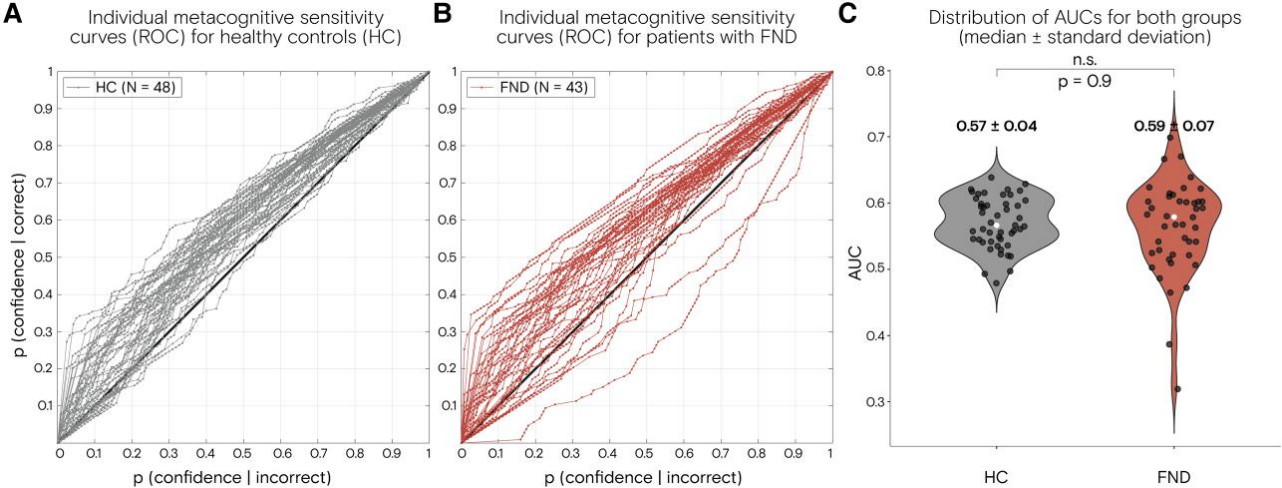
**No group differences in metacognition**. Receiver operating characteristic (ROC) curves plotted for (**A**) 43 patients with FND and (**B**) 48 HCs in the Respiratory Resistance Sensitivity Task (RRST). (**C**) No difference was detected using the Wilcoxon test for non-normally distributed data (*P* = 0.9).

Patients with FND exhibited lower respiratory sensitivity compared to HCs with a mean sensitivity for patients with FND = 2.882, SD = 1.619 and for HC = 3.527, SD = 1.128 (*P*  *=* 0.032, *d*  *=* 0.47) (Fig. 2). This group difference also remained significant when adding anxiety trait as a covariate of no interest (*P* = 0.039). In terms of interoceptive decision precision, we identified a trend towards a group difference (*P* = 0.0630), yet remarked a notable variance displayed by the patient group (FND median = 63.25, IQR = 2880.42 compared to HC median = 32.48, IQR = 49.10) (Fig. 2). No group differences in metacognition were found (*P*  *=* 0.896) (Fig. 3). Interoceptive self-report was significantly lower in the FND cohort compared to HCs (MAIA_TOTAL_; *P*  *=* 0.0004, *d* = 0.79 and IAS; *P*  *=* 0.018, *d* = 0.65). Looking at the MAIA subscale of Noticing only, there was no group difference (*P* = 0.719) (Table 2). For more details on subscales of the MAIA questionnaire, see Supplementary Fig. 1.

**Table 2:** Group differences in interoception

Variable	Overall, *N* = 91	HC, *N* = 48	FND, *N* = 43	*P*-value
MAIA_TOTAL_, mean, (SD)	22.1 (5.8)	24.1 (4.5)	19.9 (6.3)	<0.001^a^
MAIA_NOTICING_, median, (IQR)	3.25 (1.00)	3.25 (0.75)	3.25 (1.63)	0.4^b^
IAS, median (IQR)	85.0 (18.5)	89.0 (14.3)	80.0 (25.5)	0.018^b^
Respiratory sensitivity, mean (SD)	3.22 (1.41)	3.53 (1.13)	2.88 (1.62)	0.032^a^
Respiratory decision precision - RRST slope, median (IQR)	39.01 (421.44)	32.48 (49.09)	63.25 (2880.42)	0.062^b^
Respiratory metascore, median (IQR)	0.57 (0.06)	0.57 (0.05)	0.59 (0.08)	0.9^b^

^a^Welch two sample *t*-test for normally distributed data.

^b^Mann–Whitney U-test for non-normally distributed data.

TPE, trait prediction error; MAIA_TOTAL_, Mehling’s multi-dimensional assessment of interoceptive awareness total score; MAIA_TOTAL_, MAIA subscale of *Noticing*; IAS, Murphy’s interoceptive accuracy scale; SD, standard deviation; IQR, inter-quartile range.

### Interoception and somatoform dissociation

The SDQ-20 score showed a significant negative correlation with respiratory sensitivity *(r* = −0.38, *P* = 0.011) and metacognition (*r* = −0.36, *P* = 0.017) in the FND cohort, but not in HCs (respiratory sensitivity *r* = −0.04, *P* = 0.781; and metacognition *r* = 0.09, *P* = 0.530). After correction for multiple comparisons, only respiratory sensitivity and SDQ-20 remained significant (*P* = 0.045), while metacognition and SDQ-20 showed only weak evidence of a correlation (*P* = 0.070). No significant correlations were found between respiratory sensitivity or metacognition and the additional clinical metrics collected exclusively in the FND patient group (e.g. CGI and the SFMDRS; with the exclusion of the *N* = 10 that did not have motor symptoms and thus scored 0 in this evaluation). Also, between interoceptive self-reports and clinical scores for FND patients, only the IAS and the SDQ scores were correlated (*P* = 0.017), but did not survive correction for multiple comparisons (*P* = 0.208).

**Figure 4:**
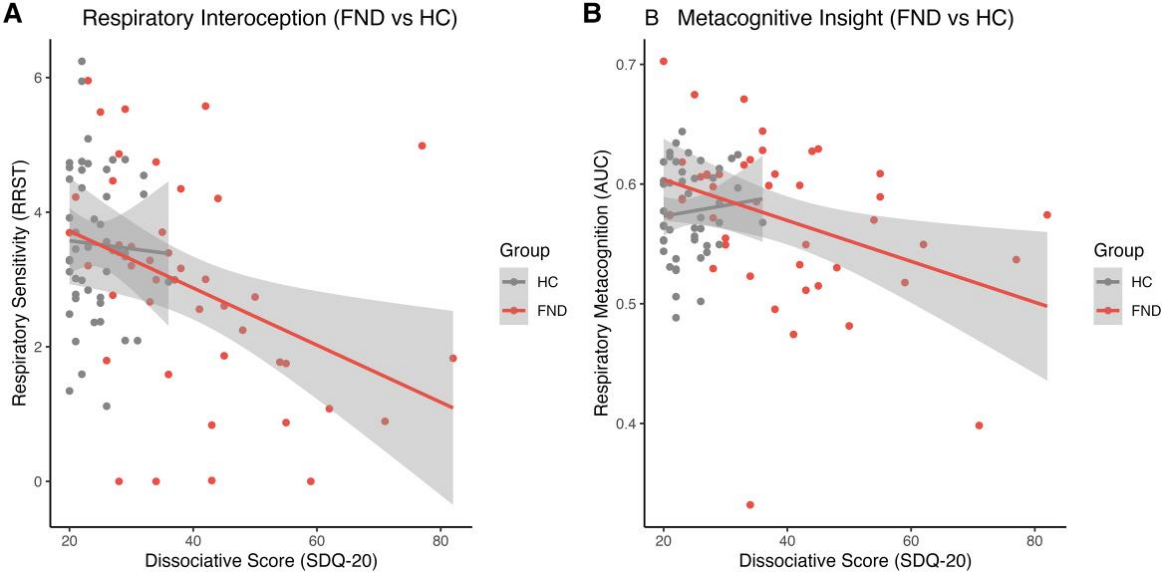
**Group-dependent interaction of dissociative scores and respiratory sensitivity or metacognition.** (**A**) In patients with FND, higher dissociation scores, measured using the SDQ-20, were associated with lower interoceptive sensitivity, measured using the respiratory resistance sensitivity task (RRST) with a Pearson’s correlation of *r* = −0.44 (*P* = 0.005). No such association was found in healthy controls (HCs) with *r* = −0.04 (*P* = 0.781). (**B**) In patients with FND, lower metascore was also associated with higher dissociative scores (*r* = −0.36, *P* = 0.017), which was again not the case for HCs (*r* = 0.09, *P* = 0.530). Metascore was operationalized as the area under the curve (AUC) of the confidence ratings in detecting obstructed breathing on the RRST. Note that there is a limited range of dissociation scores for HCs.

The multiple linear regression model with group in interaction with respiratory sensitivity, and psychotropic medication added as a covariate of no interest, was statistically significant, *F*(4,84) = 20.16, *P* < 0.001, explaining *R*^2^ = 48.98% of the variance in SDQ-20 scores. The main effect of respiratory sensitivity—representing the relationship between respiratory sensitivity and SDQ scores for the HC group—was not significant (β = 0.36, CI = −2.19–2.915, *P* = 0.778). However, the interaction between group and respiratory sensitivity was marginally significant (β = −3.10, CI = −6.22–0.026, *P* = 0.052), indicating that as respiratory sensitivity decreases; SDQ-20 scores tend to increase in the FND group relative to the HC group. This interaction effect remained marginally significant after adjusting for the effect of psychotropic medication. Psychotropic medication was also a significant predictor (β = 7.29, CI = 1.61–12.96, *P* = 0.012), suggesting higher SDQ-20 scores in medicated individuals. Figure 4A illustrates the regression slopes between respiratory sensitivity and SDQ-20 scores separately for the FND and HC groups, and the Supplemental Material presents further analysis by group.

### Metacognition and somatoform dissociation

Similarly, we investigated the association between metacognition and SDQ-20 scores within the multiple regression model, allowing for the interaction between metacognition and group, while controlling for psychotropic medication. The overall model was statistically significant, *F*(4,84) = 20.29, *P* = 1.01e-11, explaining *R*^2^ = 47% of the variance in SDQ scores. While no main effect of metascore (β = 9.52, CI = −65.27–84.31, *P* = 0.801) nor metascore interaction with group (β = −73.28, CI = −159.35–12.78, *P* = 0.094) was identified, there was a main effect of group (β = 54.34, CI = 4.55–104.14, *P* = 0.033) and the intake of medication (β = 8.00, CI = 2.51–13.49, *P* = 0.005), suggesting that FND patients and those with higher medication intake had an association with higher dissociation scores. Figure 4B shows the regression slopes between metacognition and SDQ-20 scores separately for the FND and HC groups.

### Drift rate and boundary separation

**Figure 5:**
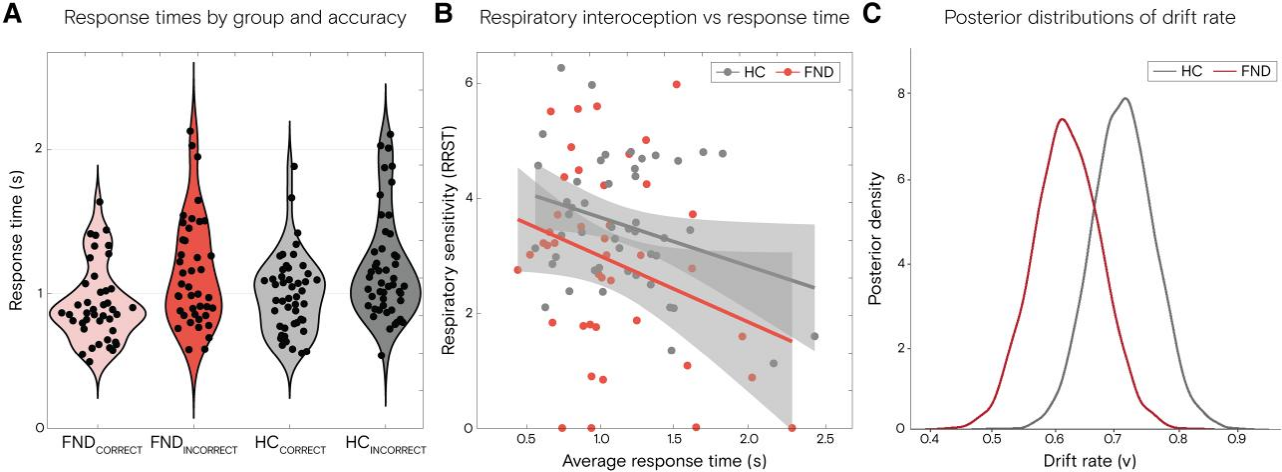
**Response time and drift diffusion in the respiratory sensitivity task**. (**A**) Violin plots demonstrating the response time separate per group (FND = 42 versus HC = 48), stratified per accuracy of detection, with no between-group difference (*P* > 0.05) and significant within-group difference (*P* < 0.001) using an independent and paired Wilcoxon test, respectively. (**B**) Interaction of RT with respiratory sensitivity for FND *r* = −0.292, *P* = 0.060, and for HC *r* = −0.30, *P* = 0.041 using Pearson correlation. (**C**) The posterior density of the drift rate, separate per group (*P*(HC > FND) = 0.89). Note that one patient was excluded from the RT analysis because she was unable to respond due to a motor impairment; the experimenter responded on the patient’s behalf.

DDM revealed that HCs had higher drift rates compared to patients with FND, with a posterior probability of 89% (*P*(HC > FND) = 0.89), suggesting moderate evidence for more efficient information accumulation in the control group (Fig. 5C). The best-fitting model also allowed boundary separation to vary by group, and there was only weak evidence that HCs adopted a more cautious decision-making strategy (*P*(HC > FND) = 0.77).

### Control for RT

There was no group difference in the average RT (FND median = 0.98 (IQR = 0.51) and HC median = 1.11 s (IQR = 0.51), *P* = 0.344). While both groups showed a faster RT for correct trials compared to incorrect trials (*P* < 0.001), no group difference was found in RT stratified for accuracy (Fig. 5A). Nonetheless, we found that average RT correlated negatively with respiratory sensitivity (*r* = −0.27, *P* = 0.013) (Fig. 5B). Thus, assessing the effect of RT on the group difference in respiratory sensitivity, we identified both group (β = −0.71, CI = −1.26–(−0.15), *P* = 0.014) and RT (β = −1.01, CI = −1.70–(−0.31), *P* = 0.005) to be associated with respiratory sensitivity (*F*(2,88) = 6.48, *P* = 0.002).

### Discomfort ratings

**Figure 6:**
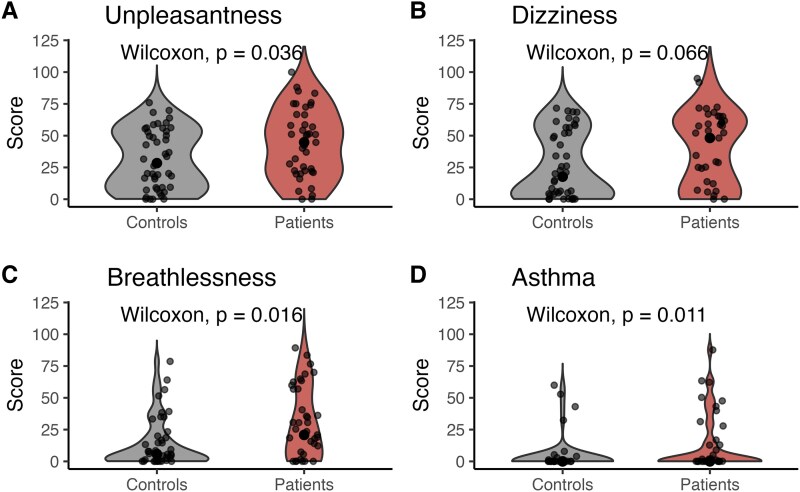
**Ratings of task unpleasantness, dizziness, breathlessness and asthma after completing the RRST separately per group.** Patients with FND (*N* = 43) showed to be more breathless at the end of the task, while showing no difference in dizziness, but reporting to have experienced more severe asthma symptoms in the week before the study visit, along with a trend to finding the task generally more unpleasant compared to controls (*N* = 48). Ratings for all control questions (**A-D)** were done from 0 = none/not at all, to 100 = completely/severe. Please note that the illustrated *P*-values are not corrected for the control of multiple comparisons, and only breathlessness remained significant (*P* = 0.007, adjusted for FDR). All comparisons were done using a Wilcoxon test, due to the non-normal distribution of the data.

The unpleasantness rating filled out at the end of the task indicated that patients with FND felt more breathless (*P* = 0.004, *d* = 0.65). None of the other ratings (unpleasantness *d* = 0.48), dizziness (*d* = 0.45) and asthma symptoms in the last week (*d* = 0.45) survived control for multiple comparison using Bonferroni. Results are shown in Fig. 6. Respiratory sensitivity was further correlated with breathlessness (*r* = −0.24, *P* = 0.026), and asthma severity in the last week (*r* = −0.33, *P* = 0.002), while neither overall unpleasantness nor dizziness were correlated with the sensitivity outcome (*P* > 0.3). Adding breathlessness or asthma severity as separate covariates to test for the group difference in respiratory sensitivity in a linear model, the group difference would disappear, indicating that the discomfort of the task is also associated with the sensitivity threshold achieved at the end. Detailed results on these control analyses can be found in the Supplementary material.

## Discussion

This study provides a novel and comprehensive examination of interoception in FND by integrating respiratory sensitivity assessment with metacognition and interoceptive self-reports. Our results demonstrate that patients with FND have reduced respiratory sensitivity in the RRST, indicating reduced interoceptive accuracy compared to HCs. The DDM further indicated that FND patients exhibited reduced efficiency in accumulating sensory evidence prior to interoceptive judgement during the task. The negative correlation between respiratory sensitivity and SDQ-20 in our FND cohort illustrates that reduced interoceptive perception is associated with a higher burden of somatoform dissociative symptoms. Finally, patients with FND also reported a lower self-reported tendency to focus on interoceptive sensations and lower self-reported interoceptive accuracy, but we did not find a difference between groups in interoceptive metacognition (e.g. the insight about their performance).

The reduced respiratory sensitivity observed in patients with FND extends previous evidence of interoceptive dysfunction and demonstrates that this impairment extends beyond cardiac interoception to include respiratory processes.^16-18,24,25^ This group difference further remains after controlling for trait anxiety scores, indicating it is not explained by the heightened level of anxiety in patients with FND. Furthermore, the severity of somatoform dissociation negatively correlated with respiratory sensitivity and respiratory metacognition specifically in the FND cohort, suggesting that interoceptive abnormalities play a role in symptom perception. Dissociation, characterized by a loss of control or awareness over typically conscious mental or somatic processes,^43^ contrasts with interoception, which integrates bodily information and is therefore central to bodily self-awareness. In FND, state dissociation such as depersonalization or derealization induced by mirror-gazing has been shown to reduce cardiac interoceptive accuracy temporarily.^20^ Moreover, in patients with functional seizures, somatoform dissociation was related to abnormal interoceptive expectations (a mismatch between interoceptive accuracy and beliefs in interoceptive ability; TPE).^16^ These sensory patterns in the cardiac and respiratory domains may reflect a tendency to detach from interoceptive signals in FND. This is consistent with observed state disruptions of interoceptive processing in FND such as reduced activation of the salience network during heartbeat counting,^22^ or reduced cortical representation of interoceptive inputs assessed via heartbeat-evoked potentials that precede functional seizure onset.^24^ Together, these findings highlight a disruption in interoception across multiple domains, potentially reflecting a core feature of FND pathophysiology. These features can also be understood in the discussed theoretical frameworks that have previously linked respiratory interoception to predictive processing relevant to neuropsychiatric disorders, and thus maybe also FND.^44,45^ Importantly, as breathing is accessible and modifiable through targeted training, respiratory interoception offers a promising avenue for therapeutic intervention.^46^

The reduced respiratory sensitivity along with the lack of group differences in respiratory metacognition suggests that FND patients were aware of their interoceptive dysfunction. The relatively intact interoceptive metacognition in our FND cohort aligns with the overall preservation of metacognition previously reported in FND.^47^ However, in our FND sample, somatoform dissociation was negatively associated with interoceptive metacognition. As symptom severity increases, it may strain cognitive and attentional resources, reducing the capacity for accurate self-monitoring. This could reflect the cognitive burden of severe symptoms, where mental resources are diverted to symptom-related processing at the expense of metacognitive monitoring, a potential mechanism also discussed in a previous review.^48^ This finding is similar to previous work showing that the interoceptive trait prediction error—defined as the difference between subjective self-reported interoceptive sensitivity and objective performance on an interoceptive task—was correlated with symptom severity (as measured by the SDQ-20), as well as with seizure frequency.^16^

The FND patients scored significantly lower on two distinct scales of interoceptive self-report. The MAIA as a total score, captures multiple dimensions of interoceptive awareness, whereas the common general factor has also been correlated to personality traits such as extraversion and openness to experience.^32^ Patients with FND reported lower total scales on this multi-dimensional interoception self-report, while the specific subscale of interest, about the *Noticing* of interoceptive signals, was not different between the two groups. Instead, in our FND cohort, we found lower scores in the subscales ‘Trusting, Self-Regulation, Body Listening, Emotional Awareness and Attentional Regulation’. Lower interoceptive self-report, as measured via the MAIA questionnaire, has been reported in patients with FND to be lower specifically for the subscale of ‘Trusting’ and ‘Not-Distracting’, indicating that patients do not trust their bodies and find it hard to distract themselves from bodily sensation.^20,23^ This reduced self-report of multi-dimensional assessment of interoceptive trait in our population may reflect either a predisposition or a consequence of the disorder—in particular, shifts in subscales assessing comfort with and confidence in one’s body and sensations may be expected in FND. The IAS, which may be less influenced by disease-related biases, has previously shown negative correlations with anxiety, depression, and somatoform symptoms in the general population^34^ and was also shown to be reduced in our FND cohort. This might highlight a new aspect of interoceptive dysfunction, in which reduced self-reported accuracy of interoceptive sensations is consistent with lower measured respiratory sensitivity.

Our results showed that patients with FND exhibited reduced drift rates during the respiratory discrimination task, indicating impaired efficiency of sensory evidence accumulation. Boundary separation—reflecting decision caution or the amount of information a person requires before taking a decision—did not differ reliably between groups. This mirrors findings by Sadnicka *et al*.,^49^ who also reported reduced drift rates but intact boundary separation during an exteroceptive tactile task. Together, these results suggest a domain-general deficit in evidence accumulation across sensory modalities in FND, rather than altered response caution. The selective reduction in drift rate supports models proposing degraded sensory input due to misdirected attention or altered predictive processing.^11^ Within a predictive coding framework, this may reflect down-weighting of sensory input in favour of overly precise prior beliefs, reducing the effective signal for decision-making. Low drift rate alongside preserved boundary separation suggests that performance deficits in FND reflect impaired sensory evidence rather than impulsivity or altered decision strategy. Our findings extend this literature by demonstrating that such impairments also affect interoceptive processing, supporting the idea that altered evidence accumulation is a transdiagnostic feature of FND.^49,50^

### Limitations

Finally, our study has several limitations. While this novel task demonstrated overall feasibility in a clinical sample of patients with FND, some participants experienced the task as challenging and tiring. The higher ratings of task discomfort found in the FND group also point in this direction. Importantly, the group difference of perceived breathlessness at the end of the task, and its negative correlation with respiratory sensitivity, must be acknowledged as a potential confounder. Nonetheless, physical discomfort and related symptoms are inherent features of the disorder, which may explain the higher subjective ratings and the associated reduction in sensitivity observed in our findings. Additionally, the externally paced breathing requirement was reported as stressful by some people, particularly those who were used to slower, deeper breathing patterns. This may have influenced performance on the breathing task in ways beyond the controlled variables. Furthermore, although we instructed participants to breathe sharply through the mouth, we did not completely restrict the airflow through the nose, so any residual airflow through the nose was uncontrolled. Generally, the absence of physiological measures (e.g. airflow, mouth pressure) can restrict our ability to fully interpret and control for the underlying mechanisms of our results. As this is the first study to compare RRST performance between groups, a priori power calculation for sample size estimation was not feasible, which is a limitation of our study and the reported results. The RRST and the analyses presented here were part of a larger, comprehensive study on interoception and its potential biological markers in FND, and the sample size was determined based on a broader study aim. Although no dedicated power analysis was conducted for the RRST, we observed a statistically significant group difference with a moderate effect size. Nonetheless, these findings should be interpreted with caution. Finally, one of the main limitations is the absence of a control task, which would have allowed us to determine whether the observed group difference is specific to interoceptive processing rather than reflecting more general cognitive or attentional impairment, fatigue, or other non-specific factors that could contribute to reduced performance in patients with FND during the RRST. Future studies with outcome specific power analysis and the inclusion of a control task will be important to replicate and build upon our presented results.

### Conclusion

In conclusion, FND patients exhibit reduced respiratory sensitivity with intact respiratory metacognition, both of which are associated with somatoform dissociation. These findings highlight the role of interoceptive deficits in the pathophysiology of FND. Importantly, respiratory interoception emerges as a promising and non-invasive therapeutic target, warranting further exploration in FND and related disorders.

## Supplementary Material

fcaf283_Supplementary_Data

## Data Availability

The complete dataset used for this study is not available according to ethical guidelines. Yet, the anonymized data of a subgroup of participants is available. All necessary information on the RRST device, along with the subgroup of participants, the scripts and statistical analyses yielding the output presented here, are publicly available on https://github.com/nstoff/RRST_FND.
