# Correction to: Respiratory sensitivity is reduced in functional neurological disorder and associated with higher somatoform dissociation

**DOI:** 10.1093/braincomms/fcaf300

**Published:** 2025-08-26

This is a correction to: Natascha Stoffel, Petr Sojka, Nicolas Gninenko, Michael Mouthon, Laure von der Weid, Tereza Serranová, Selma Aybek, Respiratory sensitivity is reduced in functional neurological disorder and associated with higher somatoform dissociation, *Brain Communications*, Volume 7, Issue 4, 2025, https://doi.org/10.1093/braincomms/fcaf283

In Table 1, the figures in the first row ('Sex, Female, Count (%)'), under the first three columns were errored and have been emended. Table 1 should read:

**Table 1:** Characteristics of sample

Variable	Overall, *N* = 91	HC, *N* = 48	FND, *N* = 43	*P*-value
Sex, female, count (%)	66.0 (72.5)	35.0 (72.9)	31.0 (72.1)	>0.9^a^
Age, years, median (IQR)	37.0 (30.0, 48.5)	37.0 (28.0, 50.0)	39.0 (31.5, 47.0)	0.6^b^
Intake of psychotropic medication, count (%)	21.0 (23.6)	2.0 (4.3)	19.0 (44.2)	<0.001^a^
Smoking, count (%)	15 (16)	6 (13)	9 (21)	0.4^a^
Body mass index in kg/m², median (IQR)	23.7 (21.7, 26.3)	22.7 (21.7, 25.1)	25.3 (21.8, 27.8)	0.019^b^
BDI-II, median (IQR)	8.0 (3.0, 15.0)	4.0 (1.0, 8.0)	15.0 (9.5, 23.5)	<0.001^b^
STAI-T, median (IQR)	41 (32, 49)	37 (30, 45)	46 (38, 55)	<0.001^b^
CTQ, median (IQR)	37.0 (30.0, 54.5)	34.0 (29.0, 41.3)	46.0 (35.0, 60.5)	0.001^b^
SDQ-20, median (IQR)	27.0 (22.0, 36.0)	23.0 (21.0, 26.0)	36.0 (28.5, 45.0)	<0.001^b^

^a^Pearson’s χ^2^ test.

^b^Mann–Whitney U-test.

BDI-II, Beck’s depression inventory II; STAI-T, Spielberger’s trait anxiety inventory; CTQ, Bernstein’s childhood trauma questionnaire; SDQ-20, Nijenhuis’ Somatoform Dissociation Questionnaire; IQR, inter-quartile range.

instead of:

**Table 1:** Characteristics of sample

Variable	Overall, *N* = 91	HC, *N* = 48	FND, *N* = 43	*P*-value
Sex, female, count (%)	25.0 (27.5)	13.0 (27.1)	12.0 (27.9)	>0.9^a^
Age, years, median (IQR)	37.0 (30.0, 48.5)	37.0 (28.0, 50.0)	39.0 (31.5, 47.0)	0.6^b^
Intake of psychotropic medication, count (%)	21.0 (23.6)	2.0 (4.3)	19.0 (44.2)	<0.001^a^
Smoking, count (%)	15 (16)	6 (13)	9 (21)	0.4^a^
Body mass index in kg/m², median (IQR)	23.7 (21.7, 26.3)	22.7 (21.7, 25.1)	25.3 (21.8, 27.8)	0.019^b^
BDI-II, median (IQR)	8.0 (3.0, 15.0)	4.0 (1.0, 8.0)	15.0 (9.5, 23.5)	<0.001^b^
STAI-T, median (IQR)	41 (32, 49)	37 (30, 45)	46 (38, 55)	<0.001^b^
CTQ, median (IQR)	37.0 (30.0, 54.5)	34.0 (29.0, 41.3)	46.0 (35.0, 60.5)	0.001^b^
SDQ-20, median (IQR)	27.0 (22.0, 36.0)	23.0 (21.0, 26.0)	36.0 (28.5, 45.0)	<0.001^b^

^a^Pearson’s χ^2^ test.

^b^Mann–Whitney U-test.

BDI-II, Beck’s depression inventory II; STAI-T, Spielberger’s trait anxiety inventory; CTQ, Bernstein’s childhood trauma questionnaire; SDQ-20, Nijenhuis’ Somatoform Dissociation Questionnaire; IQR, inter-quartile range.

These errors have been emended in the article.

