# Supplementary material for: Respiratory sensitivity is reduced in functional neurological disorder and associated with higher somatoform dissociation
Source: Brain Commun. 2025 Jul 31;7(4):fcaf283. doi: 10.1093/braincomms/fcaf283 (PMC12342147; doi:10.1093/braincomms/fcaf283)
Supplement: fcaf283_Supplementary_Data [file fcaf283_supplementary_data.pdf]

# Supplementary Material

# Respiratory sensitivity is reduced in functional neurological disorder and associated with higher somatoform dissociation

**Supplementary Table 1 Clinical Characteristics**

| Variable | Overall, N = 43 | female, N = 31 | male, N = 12 |
|---|---|---|---|
| Symptom Duration, in months, Mean (SD) | 72.2 (66.7) | 76.7 (72.0) | 60.3 (51.3) |
| Functional Dissociative Seizures, Count (%) | 8.0 (18.6) | 8.0 (25.8) | 0.0 (0.0) |
| motor + symptoms, Count (%) | 20.0 (46.5) | 13.0 (41.9) | 7.0 (58.3) |
| motor - symptoms, Count (%) | 29.0 (67.4) | 20.0 (64.5) | 9.0 (75.0) |
| sensory symptoms, Count (%) | 19.0 (44.2) | 15.0 (48.4) | 4.0 (33.3) |
| dizziness (PPPD), Count (%) | 2.0 (4.7) | 2.0 (6.5) | 0.0 (0.0) |
| cognitive symptoms, Count (%) | 4.0 (9.3) | 4.0 (12.9) | 0.0 (0.0) |

Note: patients can display more than one symptom; motor + indicates additional motor symptoms like tremor or involuntary movement, motor - indicates motor weakness or inability to move, PPPD = persistent postural-perceptual dizziness.

## MAIA subscales

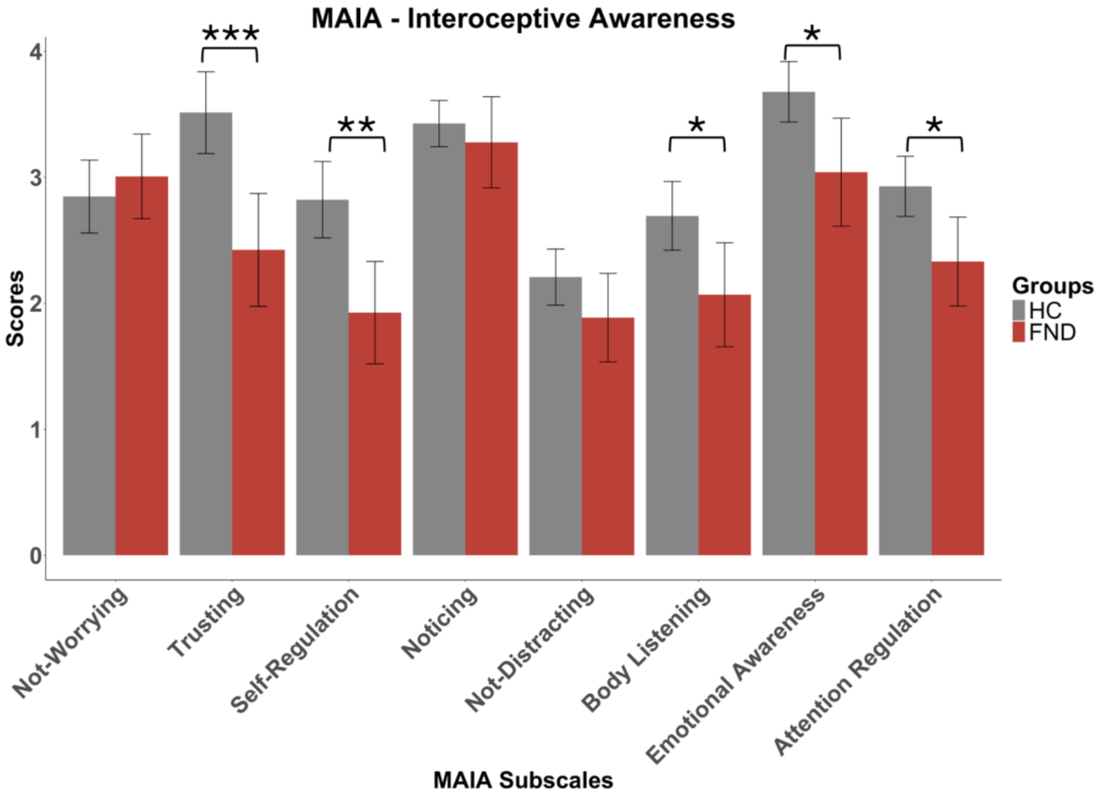


***Supplementary Figure 1. Mean scores for each MAIA subscale across groups.*** *Bars represent group means, and error bars indicate the 95% confidence intervals. A between-subject comparison was performed for each subscale using independent samples t-tests. Significant group differences are marked with asterisks (*** = $p < 0.001$, ** = $p < 0.01$, * = $p < 0.05$) based on p-values adjusted for multiple comparisons using FDR correction.*

**Control questions**

The control question consisted of the visual analogue scale, identical to the one answered for confidence trial by trial, with the following questions:

1. I find the task 0 = not unpleasant to 100 = very unpleasant
2. I feel dizzy/lightheaded: 0 = not at all to 100 = completely
3. I feel breathless/have problems breathing: 0 = not at all to 100 = completely
4. If you have asthma, rate the severity of your symptoms in the last week: 0 = no asthma/none to 100 = very severe

None of the scores on a question would correlate with another to more than $r = 0.5$, indicating that they measure differential aspects of discomfort from the task.

When including them separately in a linear regression, assessing their effect on the difference in group sensitivity, we obtained the following statistics:

Unpleasantness ratings were not associated with respiratory sensitivity ($\beta = -0.00$, CI = -0.02-0.01, $p = 0.557$) but also group was no longer related to respiratory sensitivity ($\beta = -0.56$, CI = -1.16-0.05, $p = 0.069$) for $F(2,85) = 2.25$, $p = 0.112$.

Neither breathlessness ($\beta = -0.01$, CI = -0.02-0.00, $p = 0.088$), nor group ($\beta = -0.44$, CI = -1.04-0.17, $p = 0.156$) were associated with respiratory sensitivity ($F(2,85) = 3.62$, $p = 0.031$).

And finally, for the inclusion of the asthma ratings of the last week, there was a negative association with respiratory sensitivity ($\beta = -0.02$, CI = -0.04-(-0.01), $p = 0.005$), while group was no longer significant ($\beta = -0.42$, CI = -0.99-0.16, $p = 0.153$) for $F(2,85) = 6.37$, $p = 0.003$.

**Affective Sum Score**

As clinical variables, STAI-trait and BDI-II scores were highly correlated ($r = 0.78$, $p = 2.2e-16$) in our population. Thus, we created a composite sum score from these two variables, representing affective symptoms, to prevent collinearity in the statistical models when controlling for both.

**Secondary Analysis of RRST-SDQ**

To identify which of the variables should be included in a multiple linear regression model, we tested potential variables for their influence on both the outcome variable (clinical variable; here SDQ-20) and the predictor variable (interoceptive variable; here respiratory sensitivity or metacognition) using a Pearson‘s correlation (for variables age, BMI and affective symptoms) or an independent *t*-test (for medication intake and sex). By identifying only medication as a covariate in the model, due to its association with both the SDQ-20, respiratory sensitivity and metacognition, our linear regression includes it as a covariate of no interest. Yet as a secondary analysis, the affective symptom sum score was included in the model, as it was marginally correlated with the outcome and predictor variable.

**Controlling for Affective Symptoms**

Respiratory sensitivity interacting with the group variable and controlled for the intake of psychotropic medication use and affective symptoms, was regressed from total SDQ-20 score and explained a significant proportion of the variance in SDQ score, with an adjusted $R^2$ of 0.48 ($F(5,83) = 17.08$, $p = 1.39e-11$). Within this model, only the main effect of group would have remained (β = 19.13, CI = 7.23–31.02, $p = .002$), while the main effect of medication (β = 5.05, CI = -1.14–11.23, $p = .108$), respiratory sensitivity (β =-.04, CI = -2.61–2.53, $p = .976$) and affective symptoms (β = .11, CI = -0.02–0.23, $p = .091$), as well as the interaction between respiratory sensitivity and group (β = -2.68, CI = -5.81–0.45, $p = .092$) on SDQ-20, did not remain significant. These results suggest that the effect of respiratory interoception dependent on group seems partly due to affective symptoms, or the mere group difference in SDQ-20.

Investigating the association of metacognition and group on SDQ scores within the same model (allowing for interaction and controlling for psychotropic medication and affective symptoms), we found an adjusted $R^2$ of 0.48 ($F(5,83) = 17.29$, $p = 1.09e-11$). The main effect of group would also remain significant in this model (β = 51.35, CI = 2.06–100.63, $p = .041$), while neither metacognition as a main effect (β = 6.76, CI = -67.16–80.67, $p = .856$) nor in the

interaction with group (β = -70.58, CI = -155.62–14.47, *p* = .103) was associated with a higher dissociation score, which again underlies the differences between groups on the SDQ-20, and that the higher affective symptoms cannot explain this.

**Separate analysis per group**

Regressing respiratory sensitivity on SDQ-20, while controlling for psychotropic medication for the FND cohort only, showed a marginal effect of respiratory sensitivity (β = -2.59, CI = -5.26-0.08, *p* = 0.057) and a significant effect of intake of medication use leading to higher SDQ-20 scores (β = 8.75, CI = 0.14-17.37, *p* = 0.047), F(2,40) = 5.9, $R^2$ = 18.9%, *p* = 0.006. Further adding and controlling for affective symptoms with the STAI-BDI sum score, the marginal effect of respiratory sensitivity on SDQ-20 remains (β = -2.60, CI = -5.29-0.09, *p* = 0.058), while the two covariates did not significantly add towards explaining the variance in SDQ-20 and the total model explaining $R^2$ = 17.9% of variance with F(3,39) = 4.04, *p* = 0.014.

These associations would not appear if the same analysis were performed only for the healthy control group. Only affective symptoms (β = 0.13, CI = 0.05-0.20, *p* = 0.001) were a significant predictor of SDQ-20 scores, explaining a total of R2 = 16.6% variance in SDQ-20, with F(3,42) = 4.00, *p* = 0.014.

## Confidence by Accuracy correlations

While the area under the receiver operating characteristic curve (AUROC) for measuring metacognition would represent previously used methods for measuring metacognition, some recent analyses suggest that modelling confidence-by-accuracy correlations via ordered beta regression would lead to a more sensitive and robust measure of metacognition[1]. We thank Reviewer 3 for sharing the additional code and, in acknowledgment of this contribution, present an alternative method for assessing interoceptive insight as the confidence rated as a function of stimulus intensity, response accuracy and group, **Table 2** and **Figure 2**.

**Supplementary Table 2: Predicted Confidence as a function of Stimulus Intensity, Response Accuracy, and Group**

| *Predictors* | *Estimates* | *CI Lower* | *CI Upper* | *p-value* |
|---|---|---|---|---|
| Intercept | 1.25 | 0.7 | 2.24 | 0.457 |
| Stimulus Intensity | 1.04 | 0.99 | 1.08 | 0.087 |
| Response Accuracy | 1.11 | 0.64 | 1.94 | 0.711 |
| Group (FND vs Control) | 0.66 | 0.36 | 1.19 | 0.164 |
| Stimulus Intensity × Response Accuracy | 0.95 | 0.92 | 0.99 | 0.022 |
| Stimulus Intensity × Group | 1.03 | 0.99 | 1.07 | 0.114 |
| Response Accuracy × Group | 1.3 | 0.64 | 2.67 | 0.471 |
| Stimulus Intensity × Response Accuracy × Group | 0.98 | 0.93 | 1.04 | 0.501 |
| | | | | |
| **Random Effects** | | | | |
| $\sigma^2$ | 0.14 | | | |
| $\tau 00_{\text{participamt}}$ | 0.37 | | | |
| $\tau 00_{\text{StimulusLevel}}$ | 0.04 | | | |
| ICC | 0.75 | | | |
| $N_{\text{participamt}}$ | 91 | | | |
| $N_{\text{StimulusLevel}}$ | 17 | | | |
| Observations | 4483 | | | |
| Marginal $R^2$ / Conditional $R^2$ | 0.106 / 0.775 | | | |

*Estimates are derived from a generalized linear mixed model with a beta distribution (ordbeta) for bounded outcomes (confidence ratings between 0 and 1). The model includes fixed effects for stimulus intensity, response accuracy (correct vs. incorrect), and group, as well as their interactions. Random intercepts were included for participant and stimulus intensity level.*
*CI: Confidence Interval; $\sigma^2$: residual variance; τ00: random intercept variance for participant and stimulus level; ICC: Intraclass Correlation Coefficient; N: number of levels per grouping factor; Marginal $R^2$: variance explained by fixed effects; Conditional $R^2$: variance explained by the full model (fixed + random effects).*

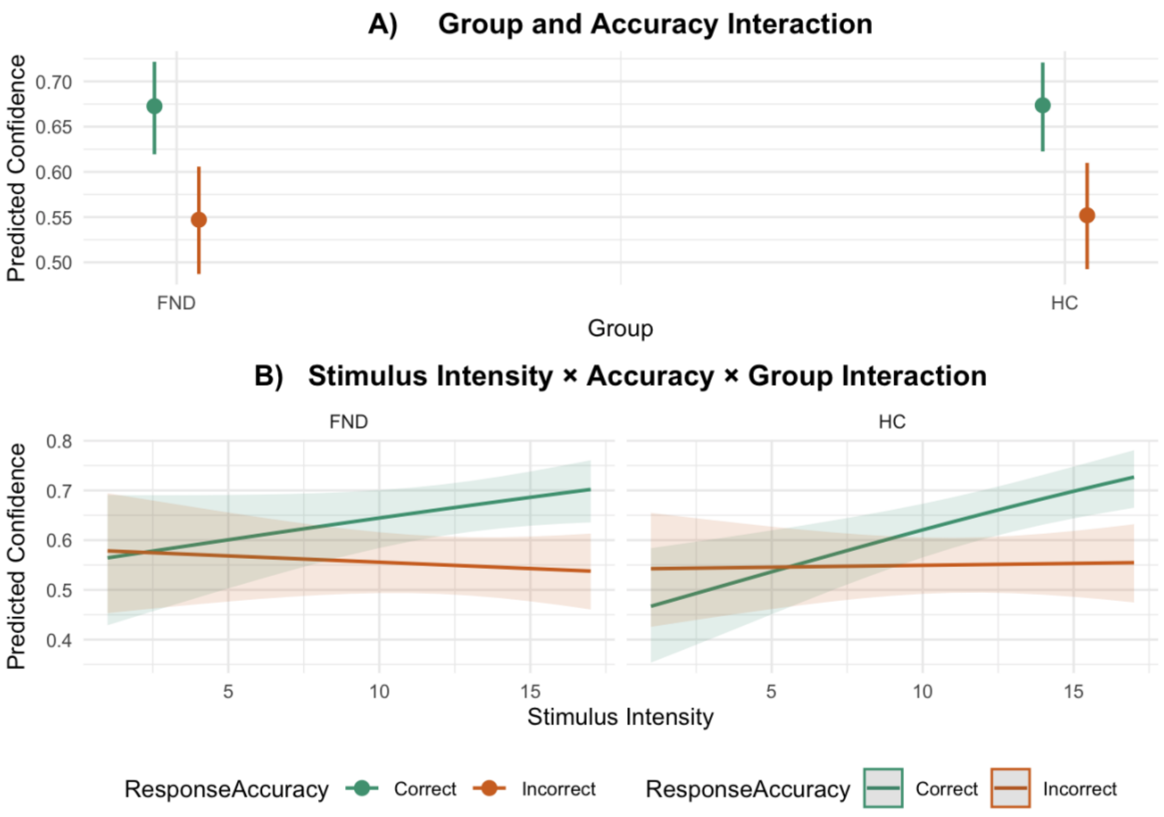


***Supplementary Figure 2. Interactions between Group, Accuracy, and Stimulus Intensity on Predicted Confidence**. A) Patients with FND reported higher confidence for correct responses compared to incorrect responses, with a significant Stimulus × Accuracy interaction (p = 0.022). B) Predicted confidence shows no increase based on stimulus intensity (*p = *0.087), neither a significent three-way interaction (Stimulus × Accuracy × Group; *p = *0.501). Predictions are based on a generalized linear mixed model fitted using glmmTMB with an ordbeta family to account for bounded continous outcomes. Colors indicate response accuracy (green = correct, orange = incorrect) and shaded bands indicate 95% confidence intervals.*

In addition to the binary variable of group, we also calculated the interaction with the clinical variable of SDQ-20, that is associated with the disorder as form of self-reported symptom severity score, **Table 3** and **Figure 3**.

**Supplementary Table 3: Predicted Confidence as a function of Stimulus Intensity, Response Accuracy, and Dissociative Symptoms**

| *Predictors* | *Estimates* | *CI Lower* | *CI Upper* | *p* |
|---|---|---|---|---|
| Intercept | 0.35 | 0.16 | 0.78 | 0.01 |
| Stimulus Intensity | 1.17 | 1.1 | 1.24 | <0.001 |
| Response Accuracy | 3.02 | 1.19 | 7.65 | 0.02 |
| SDQ | 1.03 | 1.01 | 1.06 | 0.004 |
| Stimulus Intensity × Response Accuracy | 0.86 | 0.8 | 0.93 | <0.001 |

| | | | | |
|---|---|---|---|---|
| Stimulus Intensity × SDQ | 1.0 | 1.0 | 1.0 | <0.001 |
| Response Accuracy × SDQ | 0.98 | 0.95 | 1.0 | 0.102 |
| Stimulus Intensity × Response Accuracy × SDQ | 1.0 | 1.0 | 1.0 | 0.018 |
| **Random Effects** | | | | |
| $\sigma^2$ | 0.14 | | | |
| $\tau 00_{\text{participant}}$ | 0.37 | | | |
| $\tau 00_{\text{Stimulus Level}}$ | 0.05 | | | |
| ICC | 0.75 | | | |
| $N_{\text{participant}}$ | 91 | | | |
| $N_{\text{Stimulus Level}}$ | 17 | | | |
| Observations | 4483 | | | |
| Marginal $R^2$ / Conditional $R^2$ | 0.129 / 0.784 | | | |

*Estimates are derived from a generalized linear mixed model with a beta distribution (ordbeta) for bounded outcomes (confidence ratings between 0 and 1). The model includes fixed effects for stimulus intensity, response accuracy (correct vs. incorrect), and SDQ score (Somatoform Dissociation Questionnaire), as well as their interactions. Random intercepts were included for participant and stimulus intensity level.*

*CI: Confidence Interval; $\sigma^2$: residual variance; $\tau 00$: random intercept variance for participant and stimulus level; ICC: Intraclass Correlation Coefficient; N: number of levels per grouping factor; Marginal $R^2$: variance explained by fixed effects; Conditional $R^2$: variance explained by the full model (fixed + random effects).*

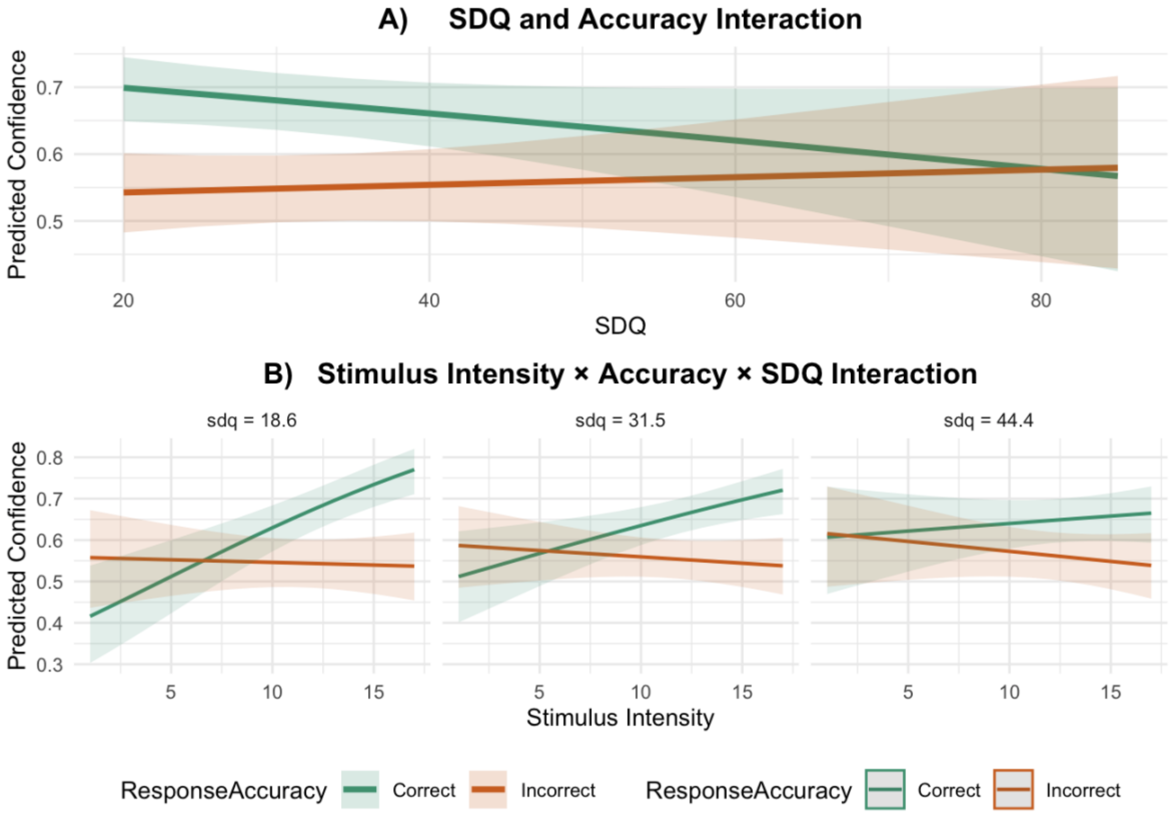


***Supplementary Figure 3.*** **Interactions between somatoform dissociative symptoms, response accuracy, and stimulus intensity on predicted confidence**. *A) Predicted confidence as a function of SDQ scores and response accuracy was fitted, showing a significant SDQ × Accuracy interaction (*$p < 0.001$*). B) Predicted confidence across stimulus intensity, response accuracy, and SDQ scores shows the increase of confidence with stimulus intensity (*$p < 0.001$*), additional to a three-way interaction between stimulus, accuracy, and SDQ (*$p = 0.018$*). Predictions were derived from a generalized linear mixed-effects model using a beta distribution for bounded confidence values (ordbeta family). Random effects were included for participant and stimulus intensity. Colors indicate response accuracy (green = correct, orange = incorrect) and shaded bands indicate 95% confidence intervals.*

References

1. Fleming SM, Lau HC. How to measure metacognition. *Front Hum Neurosci*. 2014;8. doi:10.3389/fnhum.2014.00443
